# Symbiosis dependent accumulation of primary metabolites in arbuscule-containing cells

**DOI:** 10.1186/s12870-015-0601-7

**Published:** 2015-09-30

**Authors:** Nicole Gaude, Silvia Bortfeld, Alexander Erban, Joachim Kopka, Franziska Krajinski

**Affiliations:** Max Planck Institute of Molecular Plant Physiology, Am Muehlenberg 1, 14476 Potsdam, Germany

## Abstract

**Background:**

The arbuscular mycorrhizal symbiosis is characterized by the presence of different symbiotic structures and stages within a root system. Therefore tools allowing the analysis of molecular changes at a cellular level are required to reveal insight into arbuscular mycorrhizal (AM) symbiosis development and functioning.

**Results:**

Here we describe the analysis of metabolite pools in arbuscule-containing cells, which are the site of nutrient transfer between AM fungus and host plant. Laser capture microdissection (LCM) combined with gas chromatography mass spectrometry (GC-EI/TOF-MS) enabled the analysis of primary metabolite levels,which might be of plant or fungal origin, within these cells.

**Conclusions:**

High levels of the amino acids, aspartate, asparagine, glutamate, and glutamine, were observed in arbuscule-containing cells. Elevated amounts of sucrose and the steady-state of hexose levels indicated a direct assimilation of monosaccharides by the fungal partner.

**Electronic supplementary material:**

The online version of this article (doi:10.1186/s12870-015-0601-7) contains supplementary material, which is available to authorized users.

## Background

The arbuscular mycorrhiza symbiosis (AM symbiosis) is a widespread mutualistic association. AM symbiosis involves a mutually beneficial nutrient exchange at symbiotic interfaces, in particular, phosphate and nitrogen translocation to the plant. On the other hand carbon is supplied mainly in the form of carbohydrates to the biotrophic AM fungus. During the symbiotic interaction, both partners undergo significant morphological and physiological modifications which involve alterations of the metabolite profile [1–4]. It is known that extraradical fungal hyphae take up different forms of nitrogen from the soil and transfer it to the host plant [5–8]. In addition, root carbohydrate pools are substantially altered in mycorrhizal plants [9, 10].

The AM symbiosis is characterized by the formation of highly branched structures within host cells, the arbuscules. Arbuscules are formed in the inner root cortex of mycorrhizal roots. These intracellular structures are the major site of a reciprocal nutrient transfer facilitated by a number of transporters located in the periarbuscular membrane (PAM) surrounding the arbuscules [11]. The development of the arbuscules is an asynchronous process. The distribution of all developmental stages in the root and a continual re-colonization of cortex cells complicate the analysis of specific reprogramming processes at the level of the whole root organ.

Therefore the application of single-cell isolation methods, i.e., laser microdissection, is required to analyse the cell-specific accumulation of specialized small molecules such as metabolites [12]. Single cell analysis allows the detection of potential key compounds, which have altered concentrations or become detectable only at specific developmental stages of the plant-fungus interaction. Laser microdissection was already successfully employed to investigate cell-specific alteration in RNA-, protein and metabolite levels in plants [13–21].

GC-MS is one of the most widely applied technology platform used to analyse metabolite levels. This method facilitates the identification and robust quantification of metabolites [22–24]. However, this profiling technology is often applied to whole plants or organs. As a consequence such metabolite profiles have a low spatial resolution. The knowledge of the distribution of metabolites in specific tissues or specialized plant-cells is, however, indispensable to understand the bioactive role of these molecules.

Recently, comprehensive metabolome profiling of mycorrhizal roots of barrel medic at different colonization stages was carried out by the combined application of GC-MS, HPLC and LC-MS [1]. Based on this information the combination of laser microdissection and micro-metabolomics profiling can be expected to enhance the insight into cellular metabolic processes during the symbiotic interactions with adequate spatial resolution. In this report, we address the adaptation of the plant host cells and the fungal organism to the symbiotic interaction with a specific focus on the differential accumulation of primary metabolites.

## Results and discussion

### Metabolite profiling of distinct cell types of mycorrhizal roots

In a fully developed AM symbiosis, different symbiotic structures are present in a root system. AM fungi form intracellular structures in inner cortical root cells named arbuscules, which are the site of nutrient transfer from fungus to the host plant. These cortical cells undergo a profound transcriptional reprogramming [13, 19, 25] leading to morphological and physiological changes including the development of a novel membrane type the periarbuscular membrane (PAM). Recent approaches using Laser Capture Microdissection of arbuscule containing cells revealed insights about transcriptome [19] and proteome changes [20] in these cells. Metabolome changes in mycorrhizal *M. truncatula*roots have previously been characterized at whole root level [1, 26]. However, currently no datasets about the specific metabolite composition of colonized cells are available.

Here, we combinedthe LCM technique with a gas chromatography coupled to highly sensitive time-of-flight mass spectrometry (GC-EI/TOF-MS) to investigate the metabolic changes in arbuscule-containing cells from mycorrhizal *M. truncatula* roots.

**Fig. 1:**
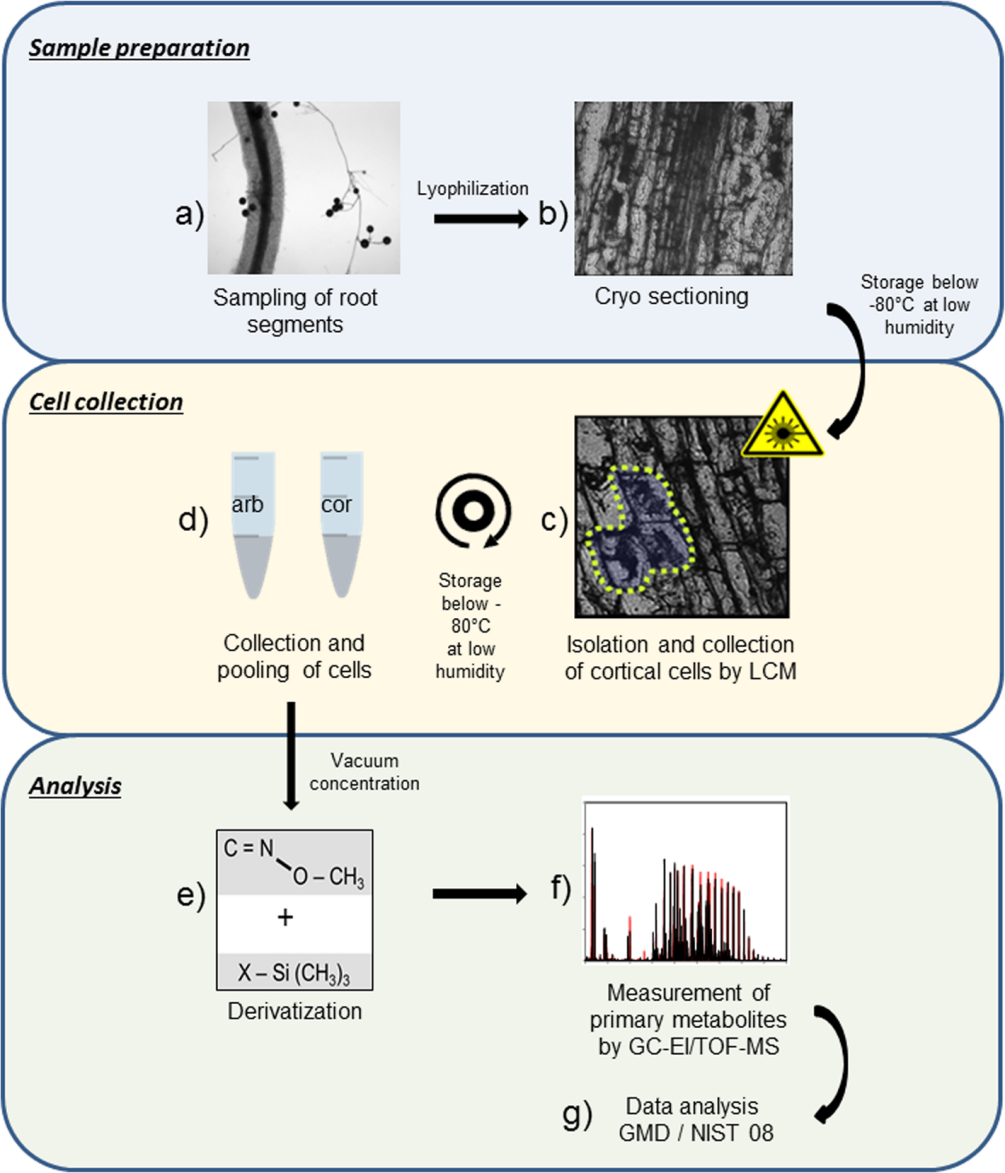
Workflow illustration: LCM-mediated harvest of root cortex cells for metabolite profiling. Root fragments of mycorrhizal and non-mycorrhizal *Medicago truncatula* plants were lyophilized and sectioned with a cryostat (**a** and **b**). In 35 μm longitudinal sections, cortical cell populations were identified and isolated by laser microdissection (**c** and **d**). Approximately 13,000 cells for each cell type (arbuscule containing cells of mycorrhizal roots [arb] and cortical cells of non-colonized roots [cor]) were collected and subjected to derivatization (**e**). GC-EI/TOF-MS measurements facilitated the abundance of primary metabolites in the analysed samples (**f**). The corresponding compounds were identified through spectral matching against the National Institute of Standards and Technology library (NIST08) (**g**)

For this purpose we used a modified a protocol originally developed for metabolite measurements in vascular bundle cells of *A. thaliana* [15] (Fig. 1). These modifications were necessary, as the highly sensitive analytical method needed a reduction of the background caused by polymeric substances originating from the fixation medium. Root fragments were longitudinally sectioned and colonized as well as non-colonized root cells were microscopically identified, cut and collected.

A microscopic image of arbuscule-containing cells, which were collected for the analyses, is shown in Additional file 1: Figure S1.

**Fig. 2:**
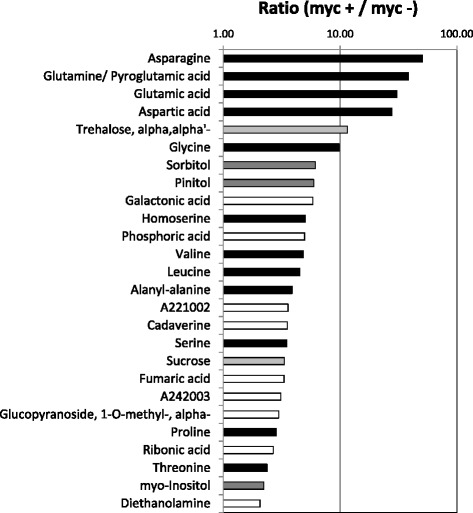
Polar primary metabolites which accumulate at least 2-fold in arbuscule containing cells of mycorrhizal roots (myc+) compared to cortex cells of non-mycorrhizal roots (myc-) of *Medicago truncatula*. Proteinogenic amino acids (black bars), polyols (grey bars) and disaccharides (light grey bars) are highlighted (cf., Additional file 2: Table S1). Yet non-identified metabolites are named by an identifier code of the GolmMetabolome Database (http://gmd.mpimp-golm.mpg.de/)

In total, 13,638 arbuscule containing cells (arb) and 12,560 cortical cells (cor) were isolated and subjected to GC-EI/TOF-MS analysis. GC-MS measurements revealed a clear difference in the metabolite composition between both cell types (Fig. 2). This indicated that GC-EI/TOF-MS-mediated analysis of primary metabolites is feasible in LMD-isolated cells and the applied method is sensitive enough to analyse limited root material.

Overall 56 annotated polar primary metabolites and 14 yet unidentified polar primary metabolites were detected in both cell populations (Additional file 2: Table S1, Additional file 3: Figure S2). The ion count ratio for each metabolite pointed to anaccumulation of distinct metabolites in arb cells (Fig. 2, Additional file 2: Table S1 and Additional file 3: Figure S2). Previously Schliemann et al. detected and quantified 81 polar compounds in roots colonized or not with *Rhizophagus irregularis* [1].

Furthermore 71 analytes were found by *Laparre* et al. to be mycorrhiza-associated [26]. In comparison to these studies it was not possible to increase the total number of detectable primary metabolites, but the application of our high-resolution method enabled the spatial information about mycorrhizal dependent accumulation of specific compounds.

LCM is a powerful tool to increase the spatio-temporal information of a given cell area or cell type [27, 28]. In recent studies, this method was successfully coupled to metabolome studies of plant tissue using GC-MS [15]. A detailed overview about handling samples for plant metabolomics has been reported in various reviews [12, 27, 29, 30]. But so far, high-resolution methods to determine metabolite changes in cells which are directly associated with the symbiotic interface are missing. Thus, the application of micro-metabolomics enables the elucidation of cell type-specific changes taking place during the arbuscular mycorrhizal symbiosis.

Although the complex chemical impurities are still detectable as background in the analysed cell populations (Additional file 4: Figure S3a), the determination of ions derived from polar primary metabolites revealed clear differences in the accumulation of these compounds. As an example, sucrose and α, αtrehalose could be detected as accumulating compounds in colonized cells in comparison to non-colonized cells after subtracting the impurities of the non-sample control (Additional file 4: Figure S3a).

A GC-EI/TOF-MS-Chromatogram depicting the absolute total ion count (TIC) of detected compounds in arbuscule containing cells and cortical cells of non-colonized roots of *Medicago* is shown in Additional file 4: Figure S3b. The count of ions in arb and cor cells was conducted in relation to the non-sample control, which consist of tissue embedding material.

#### Nutrient transfer at a cellular level

##### Most analytes in arbuscule containing cells are related to the nitrogen metabolism

All analyzed amino acids showed an accumulationin response to colonization (Additional file 3: Figure S2). Asparagine, aspartic acid as well as glutamic acid show the highest upregulation (Fig. 2). These findings are in a line with recent data showing the over accumulation of Glu, Asp and Asn for whole roots after colonization with *R. irregularis* [1]. In addition we could identify amino acids accumulating to a lower extend in arb cells. Glycine, homoserine, valine, leucine, serine, proline and threonine (Fig. 2). In summary, among the 56 identified primary metabolites in root cortex cells, 14 compounds could be classified as amino acids and all of them are upregulated in colonized root cells (Additional file 3: Figure S2).

Amino acids act as nitrogen donors, are related to stress and defense mechanisms and serve as a pool for the synthesis of other compounds. Additionally they can facilitate the nitrogen transport. Govindarajulu et al. [5] showed, that also high amounts of nitrogen are delivered to the plant during the AM symbiosis. Nitrogen is taken up from the soil by the fungal extraradical mycelium and translocated to the plant as arginine. A high proportion of the total root nitrogen originates from this symbiotic transfer [5]. Ammonium is released within the plant cells and can be assimilated to amino acids *via* the glutamine synthetase-glutamate synthase pathway (GS-GOGAT). In our transcriptomic study [19] of arbuscule containing cells we observed a high expression level of a gene encoding a glutamine synthase (mtr.36015.1.s1_at). On the other hand genes encoding nitrate reductases (mtr.10604.1.s1_at, mtr.42446.1.s1_at) are down regulated which indicates a reduced nitrate metabolism. In previous studies two putative ammonium transporters were identified, of which one (medtr7g075790.2) is induced in non-colonized cells of mycorrhizal roots cells only, whereas the other (medtr7g140920.1) is strongly induced only in arb cells [19]. Our results point to an improved NH_4_^+^ availability in the plant cell during the AM symbiosis, which is directly channelled into the amino acid synthesis. Increased amounts of asparagine, aspartic acid and glutamic acid in arbuscule containing cells are obviously associated with the higher nitrogen availability. An elevated level of arginine was not observed indicating a rapid turnover in the intraradical mycelium.

An accumulation of intermediatesof the mitochondrial tricarboxylic acid cycle (TCA) such as fumarate and succinate could be detected, whereas the level of malate, an additional compound of the TCA cycle remained unchanged (Fig. 2, Additional file 3: Figure S2). The synthesis of some amino acids such as aspartic acid and glutamic acid utilizes TCA cycle intermediates. Further, an activation of fluxes through the TCA cycle was already shown amongst others with ^13^C labelling experiments in extraradical mycelium [31] and in the intraradical mycelium [32].

##### Carbon allocation in colonized cortex cells

The most striking differences in cell specific metabolite distribution were found for those metabolites associated with nutrient transfer between plant host and fungal partner. As expected, high levels of trehalose, presumably of fungal origin, were accumulating in arb cells (Fig. 2) as already described by [1]. Trehalose is a common carbohydrate in fungi, bacteria and insects. Genes encoding for enzymes of the trehalose biosynthesis pathway were found in higher plants too, but the amount of this disaccharide is very low [33].

Recently it was shown that trehalose can act as a sucrose analogue to interfere with carbohydrate allocation. In detail, trehalose can affect carbohydrate-mediated gene expression by two hypothetical mechanisms: as an analogue of sucrose or, after cleavage, by release of glucose, and thus play a role in metabolic fine-tuning and in plant development [34, 35]. In transcriptomic studies we could already observe the high expression of sucrose regulated genes, such as sucrose synthase, specifically in arb cells [19]. These data are in line with proteomicdata analyses, were we found an accumulating sucrose synthase [20]. In our previous studies, genes directly related to the trehalose synthesis were not detected. But nevertheless, the cell specific accumulation of trehalose in arb cells might influence the metabolism in the colonized cells during the development of mycorrhizal structures by its role as signaling molecule.

We observed a strong increase of sucrose levels in arb cells. Sucrose represents the main form of carbohydrate transport in plants. In several studies, increased levels of specific sugars were observed in mycorrhizal roots [1, 36]. Saccharides either represent structural components, or serve as precursor molecules for the synthesis of a broad range of other metabolites in both, fungal and plant organisms. The fungal intraradical mycelium can take upglucose and, to a minor extent, fructose, butnot sucrose [37, 38]. *M. truncatula* mutants affected in sucrose metabolism are impaired in arbuscule development and maintenance [39]. Sucrose is cleaved by sucrose synthases, and / or invertases in the interfacial apoplast and the corresponding hexoses are taken up by the fungus. Additionally, sucrose mobilized in the cortical cells of mycorrhizal roots can serve as a carbon pool required for the synthesis of the periarbuscular membrane (PAM) in arb cells [20].

In contrast, we found only slight changes in the levels of hexoses (glucose and fructose) in arb cells when compared to cor cells. This might indicate a rapid uptake and metabolism of these hexoses by the fungus. The fast conversion of hexoses to the fungal storage compounds trehalose and glycogen was described recently [40].

**Fig. 3:**
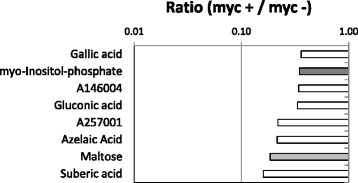
Polar primary metabolites, which are reduced at least 2-fold in arbuscule containing cells of mycorrhizal roots (myc+) compared to cortex cells of non-mycorrhizal roots (myc-) of *Medicago truncatula*. Polyols (grey bars) and disaccharides (light grey bars) are highlighted (cf., Additional file 2: Table S1). Yet non-identified metabolites are named by an identifier code of the GolmMetabolome Database (http://gmd.mpimp-golm.mpg.de/)

The reduced amounts of maltose in colonized cortex cells (Fig. 3, Additional file 3: Figure S2) might indicate a rapid breakdown to glucose by maltases.

Previous findings suggested changes in the root carbon status during mycorrhization. In detail, mycorrhizal colonization of *Lotus japonicus* roots led to a decrease in starch concentration [41]. Probably starch degradation takes place in source organs or tissues whereat maltose is formed and further cleaved to sucrose. Sucrose is transported to the root cortex cells were it is directly used for carbon supply to the fungus. Afterwards it is cleaved to glucose as this is the form of carbohydrate that is preferentially taken up by the fungus, as shown in ^14^C labeling experiments [42]. This explains the only slightly increased levels of glucose (Fig. 2), as the glucose is rapidly taken up and metabolized by the fungus.

##### Phosphate uptake in colonized cells

The phosphate uptake by the plant plays a central role in the host-fungus interaction. The increased phosphate availability, mediated by the fungal uptake and transfer, is the main benefit for the host plant [43, 44]. The phosphate content (Fig. 2, Additional file 3: Figure S2) is highly elevated in arb cells confirming the transfer of phosphate via the mycorrhizal pathway [45]. Specific plant phosphate transporters are expressed in arb cells [46–49], which mediate the uptake of phosphate from the periarbuscular space into the plant cell. This indicates that arbuscule-containing cells are the site of phosphate transfer between AM fungus and host plants. A strong increased transcript abundance of the phosphate transporter *MtPt4* and the phosphate-responsive ubiquitin-conjugating enzyme *PHO2* was detected for arbuscule and the adjacent non-colonized cells, but only at a very low level in control cells of non-mycorrhizal roots [19]. These are examples how an improved phosphate level in the colonized and adjacent cells influences the transcription of phosphate responsive genes.

#### Metabolite pattern indicates increased stress tolerance and reduced defense reactions

In the present study, significant amounts of the polyamines cadavarine, diethanolamine and to a lower extend spermidine were detectedin response to the development of mycorrhizal structures in the root cortex (Fig. 2, Additional file 3: Figure S2). Putrescine was found in arbuscule containing cells of mycorrhizal roots, but not in non-colonized root cells (Additional file 3: Figure S2). Further components related to polyamine biosynthesis were not detectable in our samples.

In general polyamines are involved in cell division and differentiation and play a beneficial role in the mycorrhizal symbiosis [50–52].

Another class of primary metabolites predominantly detected in colonized cortex cells is represented by the sugar alcohols sorbitol and pinitol. Polyols are alcohols containing multiple hydroxyl groups and their abundance often points to stress responses. As an example, pinitol accumulates during salt acclimation, but it was also reported to increase in mycorrhizal roots of *Lotus japonicus* [1, 4, 53]. Similarly, sorbitol was found to provide protection against salt stress in yeast [54] and is part of the fungal cell wall. Moreover, sorbitol is a direct product of the photosynthesis and functions as translocator of carbon skeletons and energy between sink and source organs in plants [55].

An addition, galactonic acid accumulates in arbuscule-containing cells, which can serve as a precursor for ascorbate biosynthesis. Several other organic acids, i.e. gallic acid and gluconic acid,were reduced in this cell type (Fig. 3, Additional file 3: Figure S2). Gallic acid, a soluble phenolic compound, can be found in a broad range of plant species and shows anti-fungal activity. Lattanzio et al. described the sensitivity of mycorrhizal fungi to phenolic compounds [56]. A reduction of this organic acid was detected in arbuscule containing cells indicating a repressed plant defense to accommodate the fungus. For the same reason, gluconic acid is reduced in colonized cells of mycorrhizal *M. truncatula* roots as it also function as an anti-fungal agent [57]. Gluconic acid can be produced by root-colonizing plant growth-promoting rhizobacteria (PGPR) and is a component of root exudates secreted to solubilizephosphate from the rhizosphere under phosphate limitation conditions [58]. Therefore, the improved phosphate status of a mycorrhizal plant leads to a reduction of this organic acid, too.

In the current study a reduced level of azelaic acid, a dicarboxylic acid, was determined in arb cells (Additional file 3: Figure S2). This defense compound confers resistance to pathogenic bacteria by priming the systemic plant immunity [59]. Although no antifungal activity was observed *in vitro* for azelaic acid [59] root cortex cells might decrease the amounts of azelaic acid to accommodate the mycorrhizal fungus.

## Conclusions

A combination of cryosectioning and LCM for cell isolation, followed by GC-EI/TOF-MS as analytical tool, enabled us to determine changes in primary metabolite distribution as a result of the colonization of *M. truncatula* roots by the AM-fungus *R. irregularis*. These changes in metabolism strongly suggest a symbiosis-dependent adaptation of the colonized cortical root cells to accommodate the fungal partner.

Furthermore, the here developed protocol for the analysis of *M. truncatula* roots is a reliable and valuable tool for further investigations, e.g., of the lipophilic and secondary metabolites in colonized mycorrhizal cells from Medicago roots.

## Methods

### Plant material and growth conditions

*Medicago truncatula* Gaertn. cv. Jemalong (A17) seeds were scarified in concentrated H_2_SO_4_ for 8 min, rinsed 8–10 times with water and surface sterilized with 6 % sodium hypochlorite for 10 min. The seeds were distributed on wet filter paper in Petri dishes. Stratification was carried out at 4 °C overnight. The seeds were incubated for further 4 days at 25 °C, in the dark. The seedlings were planted in to pots containing a mixture of clay, silica sand, vermiculite and inoculum with *Rhizophagus irregularis* (30 % *v/v*) as a substrate. Inoculum was obtained by pre culturing of *Allium schoenoprasum*with *Glomus intraradices*. The plants were grown under controlled environmental conditions in a greenhouse for 21 days at 24 °C with a 16-h-light / 8-h-dark cycle. Plants were fertilized with 0.5 × Hoagland solution [60] containing 20 μM phosphate, twice per week.

### Protocol for Laser Capture Microdissection (LCM)-mediated collection of cortical cells for metabolite profiling

Scalpel dissected root fragments (1 cm long) of mycorrhizal (21 dpi) and non-mycorrhizal *Medicago truncatula* plants were snap-frozen in liquid N_2_and thoroughly dried by 12 h lyophilisation (ALPHA 2–4, Christ, Osterode, Germany) to inhibit metabolite changes due to enzyme activity and the loss of solutes. To avoid excessive contaminations by embedding material (Tissue freezing medium, Electron Microscopy Sciences, Hatfield, USA), the lyophilized root pieces were arranged on the top of a frozen embedding material block and glued by a short touch of the embedding block with a finger. Longitudinal sections (30 μm thick) were cut by using a cryostat at −22 °C (Leica CM 1950 Cryostat, Leica Microsystem, Wetzlar, Germany). Uncoated glass slides (Super frost, VWR, Darmstadt, Germany) were washed with ethanol (Merck, Darmstadt, Germany) and after drying with root sections assembled. The P.A.L.M. Laser Microbeam System (Bernried, Germany) was utilized for microdissection of cortical root cells. The following parameters were selected using P.A.L.M.Robosoftware 2003 (Microlaser Technologies, Bernried, Germany): auto-LPC focus, with energy of 95 and a speed of 100 (system specific units). The cutting and isolation was done under manual supervision with a low heat UV (337 nm nitrogen) laser at 40-fold magnification.

All consumables (tips and reaction tubes) were washed twice with ethanol and dried. Isolated specimens were directly catapulted in a lid of 0.5 ml reaction tubes with adhesive caps (Carl Zeiss Microimaging, Göttingen, Germany). Thirteen thousand six hundred thirty-eight arbuscule containing cells of mycorrhizal roots and 12,560 cortical cells of non-colonized roots were collected for subsequent metabolite analysis. The cell volume of each single dissected sample was estimated by the LCM software package by using the P.A.L.M. Robosoftware 1.2 (Microlaser Technologies, Bernried, Germany) and sum up to calculate the total cell volume.

### Metabolite extraction and profiling

Polar primary metabolites of the cell type-specific populations obtained by laser capture microdissection from lyophilized *Medicago truncatula* roots were analyzed by gas chromatography-electron impact ionization/ time of flight-mass spectrometry (GC-EI/TOF-MS). Due to the high concentration factor required after extraction and because of the contact of the sample with embedding material and sampling tubes with adhesive caps, contaminations by respective laboratory chemicals were expected. To distinguish between contaminants and the endogenous metabolites of the collected single cell population’s non-sample control experiments were performed with empty LCM-tubes. The tubes were either filled with 300 μl ethanol or with a mixture of 300 μl ethanol and 1 mm^3^ embedding material and processed in parallel with the regular samples. Cells isolated by laser capture microdissection were collected at the bottom of the tube by vigorously shaking in 300 μl ethanol and centrifugation for 2 min at 20.000 g. The samples were dried by vacuum centrifugation (Concentrator 5301, Eppendorf, Hamburg, Germany) without removing residual solid material of the cuttings and stored at −80 °C for up to 3 months in the presence of silica balls. The dry pellet was dissolved in 3 μl freshly prepared methoxamine hydrochloride dissolved at 40 mg/mL in pure pyridine and incubated at 30 °C for 90 min, followed by derivatization with 9 μl of a mixture consisting of *N*-methyl-*N*-(trimethylsilyl)-trifluoroacetamide (MSTFA) and a time standard (C_10_, C_12_, C_15_, C_18_, C_19_, C_22_, C_28_, C_32_ and C_36_n-alkane mixture) at 37 °C for 30 min [61]. Care was taken to avoid humidity throughout the process. The alkanes added to the samples were used to calculate retention time indices (RIs) [62].

The samples were analysed immediately after chemical derivatization by GC-EI/TOF-MS profiling using an Agilent 6890 N gas chromatograph (Agilent Technologies, Böblingen, Germany) with splitless injection onto a FactorFour VF-5 ms capillary column, 30 m length, 0.25 mm inner diameter, 0.25 μm film thickness (Varian-Agilent Technologies), which was connected to a Pegasus III time of flight mass spectrometer (LECO Instrumente GmbH, Mönchengladbach, Germany) as described by [61, 63]. Two technical replicates of each sample were conducted.

### Data analysis

GC-EI/TOF-MS chromatograms were baseline corrected and exported in NetCDF file format using ChromaTOFsoftware (Leco, St. Joseph, USA). Primary metabolites were identified manually supervised by differential analysis compared to the non-sample controls and subsequent spectral and retention index matching against the reference collections of the Golm Metabolome database (http://gmd.mpimp-golm.mpg.de/) [64–66] and of the National Institute of Standards and Technology (NIST 08; http://www.nist.gov/srd/nist1a.cfm). Guidelines for manually supervised metabolite identification were the presence of at least 3 specific mass fragments per compound and a retention index deviation <1.0 % [62].

Peak heights of observed mass features were extracted from the NETCDF files and aligned according to retention index and processed into a standardized numerical data matrix using the TagFinder software [67]. Background subtraction of peak heights was performed, if required, using the average peak height of non-sample controls. Normalized responses were calculated, dividing by total estimated cell volume of the pooled micro-dissected samples (using LCM software package P.A.L.M. Robosoftware 1.2, Microlaser Technologies, Bernried, Germany) and dividing by peak height of the internal standard, docosane. Docosane was absent from the non-sample controls. The normalized response of a metabolite in the arbuscular cell preparation was divided by the normalized response of the metabolite in the cortex cells.

## Additional files

Additional file 1: Figure S1. Example of a colonized area used for the sample collection via LCM. Longitudinal cryosections (35 μm) of roots 21 day post infection with *Rhizophagus irregularis* or control non-mycorrhizal roots were used for cell sampling. As an example, arbuscule-containing cells (arb) collected for this study are highlighted with yellow dashed lines. (PPT 849 kb) Additional file 2: Table S1. Summary of polar primary metabolites identified in root cortical cells of mycorrhizal and non-mycorrhizal roots of *M. truncatula*. Polar metabolites were analysed by GC-EI/TOF-MS based on metabolic profiling of methoxyaminated and trimethylsilylated metabolites. Data are expressed as means of two technical replicates. (XLS 57 kb) Additional file 3: Figure S2. Polar primary metabolites which were detectable in arbuscule containing cells of mycorrhizal roots (myc+) and in cortex cells of non-mycorrhizal roots (myc-) of *Medicago truncatula* or in either of the cell types. Metabolites are sorted within class according fold change. Yet non-identified metabolites are omitted (cf., Supplemental Table S1). Mean centered normalized responses and the respective response ratios are shown. (PPT 173 kb) Additional file 4: Figure S3. GC-EI/TOF-MS-Chromatograms of a primary metabolite fraction that was extracted from cell populations of ~ 13,000 arbuscule containing root cells (red) after laser capture dissection compared to an extract from an approximately equal number non-colonized cortical cells of *Medicago truncatula* (black). (A) Selected ion monitoring using specific and selected mass fragments allows the highly selective relative quantification of metabolites in highly complex preparations that may contain unavoidable chemical contaminations. The chosen mass fragment, m/z = 361, allows the specific analysis of sucrose and α,α-trehalose at retention times that were determined by pure authenticated reference compounds. The insert shows the α,α-trehalose peak compared to a non-sample control (grey). The arrow indicates one of the impurities that need to be eliminated from further analysis by background subtraction. (B) Total ion chromatogram (TIC) of the same samples exemplifies the complex chemical impurities, which result from the embedding material that is required for the laser capture dissection process. Note, one mycorrhization-responsive compound, i.e., asparagine (red arrow), was already directly detectable by differential display analysis of the TICs. (PPT 1315 kb)

## Abbreviations

AM symbiosis: Arbuscular mycorrhizal symbiosis
arb: Arbuscule containing cells of mycorrhizal roots
cor: Cortical cells of non-mycorrhizal roots
GC-EI/TOF-MS: Gas chromatography–mass spectrometry, HPLC, High performance liquid chromatography
LCM: Laser Capture Microdissection
LC-MS: Liquid chromatography-mass spectrometry
myc+: Mycorrhizal roots
myc-: Non-mycorrhizal roots
TIC: Total ion chromatogram
