# Supplementary figures and images for: Symbiosis dependent accumulation of primary metabolites in arbuscule-containing cells
Source: BMC Plant Biol. 2015 Sep 30;15:234. doi: 10.1186/s12870-015-0601-7 (PMC4590214; doi:10.1186/s12870-015-0601-7)

## Slide 1
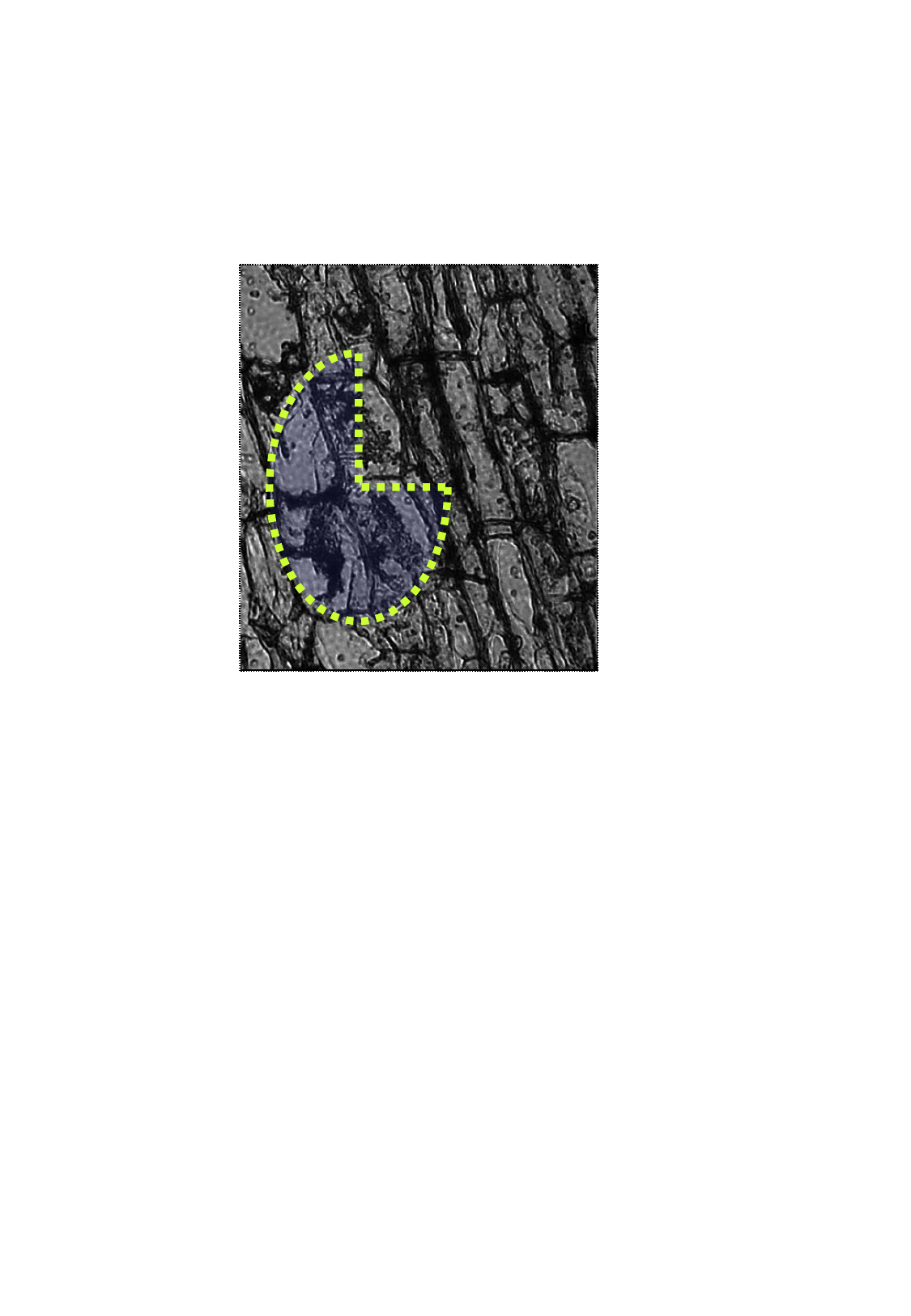

Supplement: Additional file 1: Figure S1. — Example of a colonized area used for the sample collection via LCM. Longitudinal cryosections (35 μm) of roots 21 day post infection with Rhizophagus irregularis or control non-mycorrhizal roots were used for cell sampling. As an example, arbuscule-containing cells (arb) collected for this study are highlighted with yellow dashed lines. (PPT 849 kb) [file 12870_2015_601_MOESM1_ESM.ppt]

## Slide 1
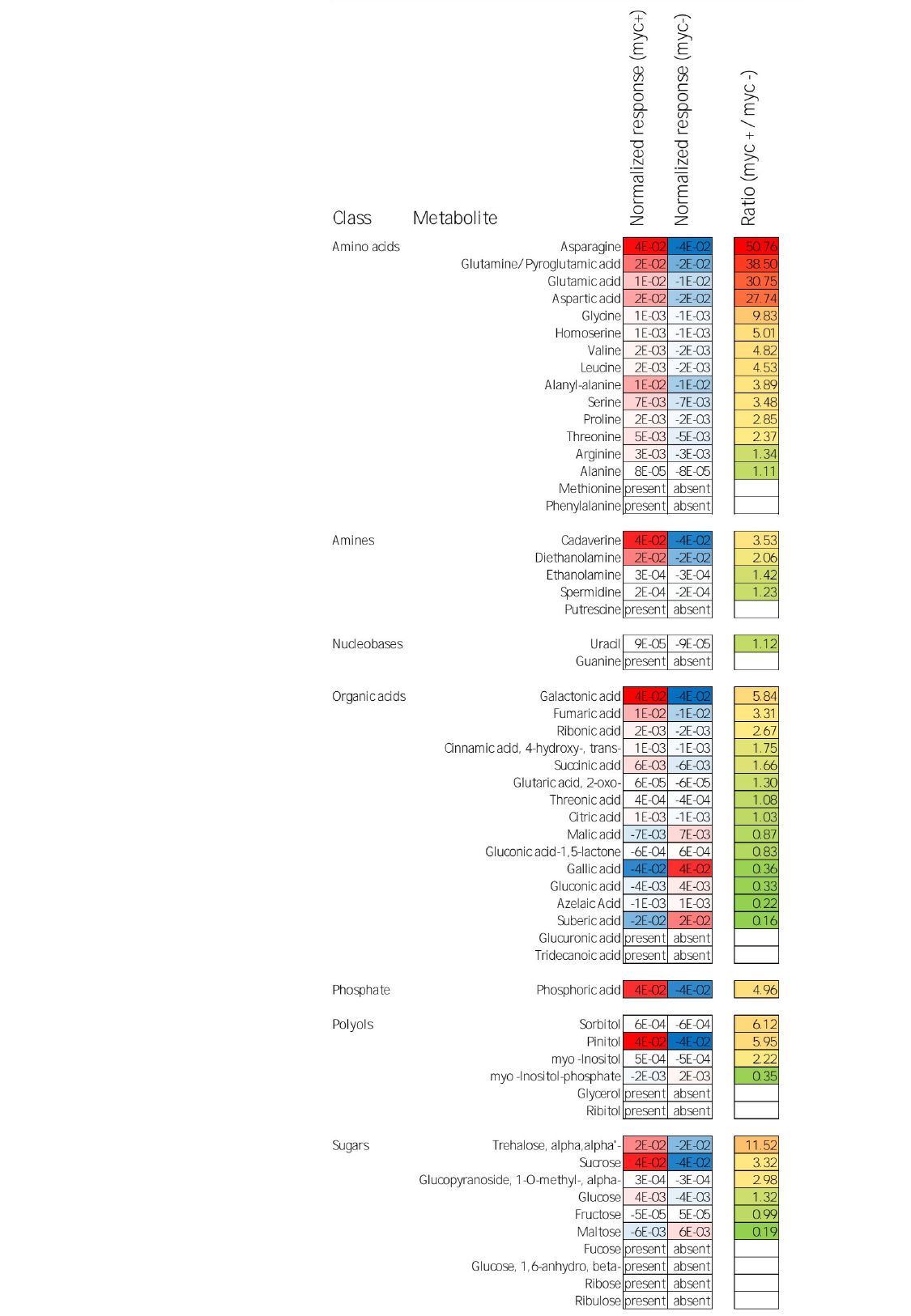

Supplement: Additional file 3: Figure S2. — Polar primary metabolites which were detectable in arbuscule containing cells of mycorrhizal roots (myc+) and in cortex cells of non-mycorrhizal roots (myc-) of Medicago truncatula or in either of the cell types. Metabolites are sorted within class according fold change. Yet non-identified metabolites are omitted (cf., Supplemental Table S1). Mean centered normalized responses and the respective response ratios are shown. (PPT 173 kb) [file 12870_2015_601_MOESM3_ESM.ppt]
