# Supplementary material for: Symbiosis dependent accumulation of primary metabolites in arbuscule-containing cells
Source: BMC Plant Biol. 2015 Sep 30;15:234. doi: 10.1186/s12870-015-0601-7 (PMC4590214; doi:10.1186/s12870-015-0601-7)
Supplement: Additional file 4: Figure S3. — GC-EI/TOF-MS-Chromatograms of a primary metabolite fraction that was extracted from cell populations of ~ 13,000 arbuscule containing root cells (red) after laser capture dissection compared to an extract from an approximately equal number non-colonized cortical cells of Medicago truncatula (black). (A) Selected ion monitoring using specific and selected mass fragments allows the highly selective relative quantification of metabolites in highly complex preparations that may contain unavoidable chemical contaminations. The chosen mass fragment, m/z = 361, allows the specific analysis of sucrose and α,α-trehalose at retention times that were determined by pure authenticated reference compounds. The insert shows the α,α-trehalose peak compared to a non-sample control (grey). The arrow indicates one of the impurities that need to be eliminated from further analysis by background subtraction. (B) Total ion chromatogram (TIC) of the same samples exemplifies the complex chemical impurities, which result from the embedding material that is required for the laser capture dissection process. Note, one mycorrhization-responsive compound, i.e., asparagine (red arrow), was already directly detectable by differential display analysis of the TICs. (PPT 1315 kb) [file 12870_2015_601_MOESM4_ESM.ppt]

## Slide 1
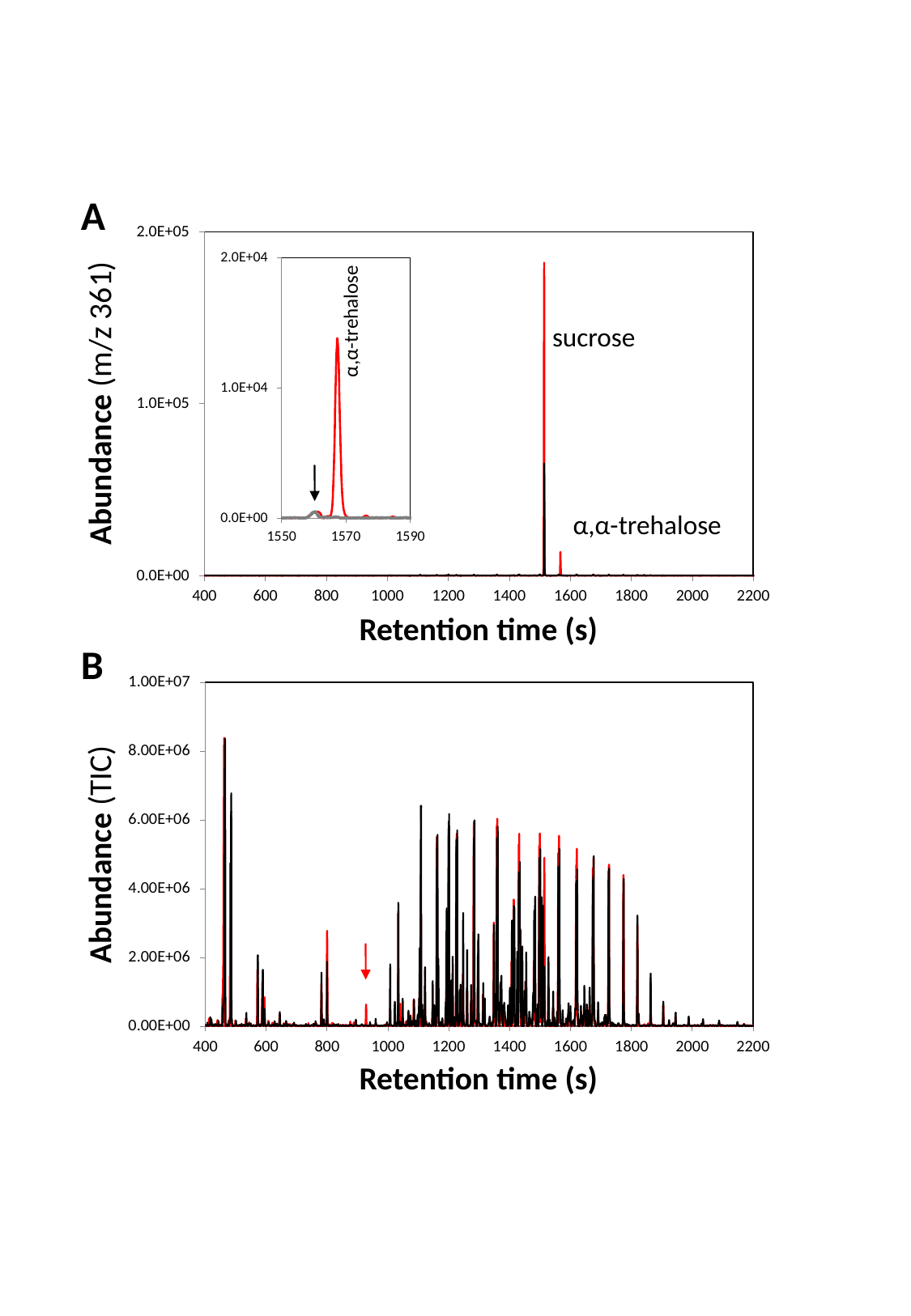

A
α,α-trehalose
sucrose
Abundance (m/z 361)
α,α-trehalose
Retention time (s)
B
Abundance (TIC)
Retention time (s)
